# The viewpoints of residents of Kerman, Iran regarding the challenges and barriers of preparing households against earthquakes: A theory-guided qualitative content analysis

**DOI:** 10.3389/fpubh.2022.1036311

**Published:** 2022-11-24

**Authors:** Esmat Rezabeigi Davarani, Hojjat Farahmandnia, Narges Khanjani, Mahmood Nekoei-Moghadam

**Affiliations:** ^1^Health in Disasters and Emergencies Research Center, Institute for Futures Studies in Health, Kerman University of Medical Sciences, Kerman, Iran; ^2^Environmental Health Engineering Research Center, Kerman University of Medical Sciences, Kerman, Iran

**Keywords:** earthquakes, family, risk reduction behavior, psychological theory, barriers

## Abstract

**Introduction:**

Earthquakes cause a lot of damage and casualties. For various reasons, most households are not prepared for earthquakes. This study aims to identify the challenges and barriers to households' preparedness against earthquakes from the viewpoint of Kerman residents.

**Methods:**

This qualitative-directed content analysis study was conducted from December 2021 to May 2022 in the city of Kerman in southeast Iran. Data was collected by purposive sampling through in-depth and semi-structured individual face-to-face interviews with 48 households.

**Results:**

After multiple rounds of analyzing and summarizing the data based on the social-cognitive theory and taking into consideration similarities and differences, five main categories and 19 subcategories created based on the results of data analysis and including (1) Challenges related to cognitive factors (2) Challenges related to behavioral factors (3) Challenges related to the physical environment (4) Challenges related to the social environment and (5) Challenges related to financial factors.

**Conclusion:**

Although the participants listed many challenges and barriers in different fields, in order to overcome the barriers and challenges of preparing households for an earthquake, the support of the authorities and the cooperation of the residents are necessary.

## Introduction

Earthquakes cause extensive damage to homes, businesses, and infrastructure, as well as casualties and financial losses (1). As the World Health Organization (WHO) reported, over the past century alone, 1,150 fatal earthquakes have happened in 75 countries worldwide (2). Earthquakes annually cause more than 10,000 deaths, most of which occur in developing countries (3). Iran is one of the most seismic regions in the world, and many destructive earthquakes have occurred in this country (2). About 93% of regions in Iran are at risk of earthquakes. More than 70% of Iran's big cities are located near active faults (4). Iran has experienced 18 earthquakes of over seven Richter magnitudes in the past 90 years, causing severe socioeconomic damage and killing thousands (3). In Kerman province alone, more than 40,000 people have died from earthquakes in recent years (5).

Disaster risk reduction management consists of three phases. Prevention, mitigation, and preparation are in the phase before the occurrence of a hazard; response and emergency relief are in the phase during the occurrence of a hazard; and recovery (reconstruction and rehabilitation) is in the phase after the hazard (6). Acquiring knowledge, skills, planning, and storing emergency equipment and supplies are essential measures in the preparation phase (7). According to studies, most households are not prepared for hazards (8–11). Preparedness against hazards is affected by various factors, including demographic, behavioral, environmental, social, cognitive, economic, physical, and cultural factors (7, 8, 11–14). From the point of view point of the social cognitive theory, neither individual factors nor environmental stimuli alone can affect a person's behavior. One of the essential features of this theory is the combination of social structures with personal dimensions (15).

Studies in different parts of the world have shown that perceived challenges and barriers significantly reduce preventive measures and disaster preparedness (10, 16–19). In a study by Najafi et al., conducted after the Kermanshah earthquake, earthquake victims commented on the necessities and challenges of earthquake preparedness in various fields (20).

Considering that the present research is a part of an exploratory sequential study, it was necessary to identify the opinions and beliefs of people about the barriers and challenges of households' preparedness against earthquakes for formulating items and designing tools. From the viewpoint of Kerman residents, since Kerman province is one of the earthquake-prone regions of Iran and most of the people in this region have experienced earthquakes, this study aims to identify the challenges and barriers to households' preparedness against earthquakes.

## Methods

### Study design

A qualitative study was conducted from December 2021 to May 2022 as part of more extensive research on the design and validation of a tool for determining the factors influencing households' preparedness behaviors for earthquakes based on the social-cognitive theory. This study was conducted using a directed content analysis approach. When there is a theory or research about a phenomenon, but it is not complete and more descriptions should be made about it, the researcher uses a directed content analysis approach (21). Pre-existing theory can help to design interview questions.

### Study population, participant selection, and data collection

The research population was the households of Kerman city. The participants were selected using a purposive sampling method and with maximum diversity in terms of gender, age, education, occupation, region of residence, experience or lack of experience of destructive earthquakes, and the presence of vulnerable groups at home. Samples were selected from four health centers in different parts of Kerman city. Participants were selected with the cooperation of healthcare workers. First, the health workers contacted the heads of households, and after stating the research objectives, they obtained their consent to participate in the interview. Then the researcher was introduced to the participants. Because the responsibility of preventive measures and preparation against earthquakes rests with the father or mother of the family, one of them participated in the interview at their own discretion. Before the interview, the purpose of the interview was explained. The participants were assured that their information would be recorded confidentially and anonymously.

Data was collected through in-depth individual semi-structured interviews. All interviews were conducted face-to-face. The participants themselves decided the time and place of the interview. The interview was conducted with open and predetermined questions. The questions about the obstacles and challenges of households' preparedness against earthquakes were based on Bandura's social-cognitive theory constructs. Before the interview started, the study's objectives were explained. Participants were assured that their participation in the study was voluntary and that the information would remain confidential. Informed consent was obtained from all participants for the interview and audio recording. Before the interview, a brief explanation of the concept of cognitive, behavioral, and environmental factors was given to the participants. For example, it was explained to them that the barriers of the physical environment include problems related to the building, the items inside the building, the furniture, the neighborhood environment, etc. The obstacles and challenges of the social environment, including the problems that the members of society have in their relationships with each other, or the obstacles that are related to the interaction between people and groups, institutions, or government organizations, each of these obstacles can affect their readiness to play a role in earthquakes.

Each interview began with an open question, and during the interviews, exploratory questions such as “What do you mean by this?” or “Could you please explain more?” were used. The interview questions are presented in Table 1. Data saturation was achieved after 48 interviews. After interviewing 46 households, the researcher came to the conclusion that saturation had been achieved and stopped sampling after conducting two more interviews to ensure that no new data was obtained. Due to the disagreement of 12 participants with recording interviews, note-taking was used instead of recording for those 12 samples. Interviews lasted between 35 and 60 min.

**Table 1:** Open-ended questions.

**Factors**	**Questions**
Cognitive barriers and challenges	1- What barriers or challenges exist to increasing households' knowledge to prepare against earthquakes? 2- What false beliefs do people have that make them not take action to prepare against an earthquake? 3- What are the negative consequences of preparing for earthquakes that causes households not ready for it? 4- What beliefs do people have about their abilities that barrier earthquake preparedness measures?
Behavioral barriers and challenges	1- What inappropriate and wrong behaviors are there among the people before, during and after the earthquake?
Environmental barriers and challenges	1- What are the barriers and challenges in the physical environment that affect the preparedness of families against earthquakes? 2- What are the barriers and challenges in the social environment that affect the preparedness of families against earthquakes?

### Inclusion and exclusion criteria

Willingness to participate in the research, having time, and spending the necessary time interviewing were included. The father or mother of the family, whoever was more inclined to participate or could provide more information, was interviewed. Households that did not have points to express or did not want to be interviewed, and households where the couple's age was <18 years old were excluded.

### Analysis and presentation

Data analysis was performed according to the steps proposed by Hsieh and Shannon (21). Data analysis was done simultaneously with data collection. The data was analyzed using the directed content analysis method. According to this approach, the key concepts of social-cognitive theory were considered as the first category. Then practical definitions for each category were determined using the structures of this theory. First, the content of the recorded or noted interviews was typed verbatim. The text was reviewed several times to find content that matched the predefined categories. Sentences and phrases were considered as meaning units. Coding of the interview text was done based on pre-specified features, and the codes were placed in the identified categories based on conceptual similarity. Categories and subcategories were formed depending on the scope and logical relationships of the data. Also, the codes that did not correspond to the initial predefined categories were defined as new categories.

### Data accuracy and robustness

This study employed strategies recommended by Lincoln and Guba for assuring trustworthiness (22). To obtain credibility, the researcher had sufficient and appropriate interaction with the participants and gained their trust to collect information. Moreover, sufficient time was allocated for data collection and analysis (6 months). Also, the participants from several health centers were selected with maximum variety. Peer-check strategies assess the dependability of data. Peer-check was performed monthly to ensure that the research team had a thorough discussion about the data. Also, member-checking by two participants and rectifying the codes that did not accurately describe the participants' point of view (based on their own opinion) improved dependability. To improve the confirmability, the supervisor and advising professor reviewed some quotations, codes, and extracted categories and confirmed the accuracy of the coding process. For data transferability, the study results were given to several people who had the same characteristics as the project participants, but did not participate in the study (external check), and they were asked to state whether they agreed with the project results.

## Results

A total of 48 interviews were conducted with 48 participants (21 men and 27 women). The average age of the participants was 42 years. The average size of the household was about four people. Nine households lived in a rented house. Twenty-four households lived in apartments. Forty-two participants had academic education. There was a vulnerable person in 37 households. Five hundred and six codes were identified without calculating the overlaps. After multiple rounds of analyzing and summarizing the data based on the social-cognitive theory and taking into consideration similarities and differences, five main categories and 19 subcategories created based on the results of data analysis. Main categories including (A) Challenges related to cognitive factors (B) Challenges related to behavioral factors (C) Challenges related to the physical environment (D) Challenges related to the social environment and (E) Challenges related to financial factors shows in Table 2.

**Table 2:** Categories, subcategories, and codes about preparing households against earthquakes based on Bandura's social cognitive theory.

**Categories**	**Subcategories**	**Example of the codes**
Challenges related to cognitive factors	Lack of Knowledge	Impossibility of using educational materials (low literacy, illiteracy, old age) Reluctance to participate in training Lack of attractiveness of training Inadequacy of training Lack of time to attend training Lack of access to face-to-face and practical training The information propagated by social networks might be misleading.
	False Beliefs	Fatalism Misconceptions about the origin of earthquakes Believing that if they think too much about earthquakes, it will happen.
	Self-efficacy	Lack of belief in their ability to use tools and equipment Lack of belief in their ability to maintain calmness and appropriate behavior during an earthquake Lack of belief in their ability to learn and use the training Lack of belief in their ability to help others during an earthquake
	Negative outcome expectations	Having fear and stress during training and exercise Thinking that children have lack of understanding about preparation and drills and there is a possibility of psychological and physical harm to them The possibility of causing physical and mental injuries in the elderly, disabled and pregnant women due to movement restrictions and stress during exercise
Challenges related to behavioral factors	Lack of skills	Lack of skills in the correct arrangement of home furniture Lack of fire extinguishing skills at home Lack of first aid skills and ability to resuscitate patients Lack of skill in cutting off electricity and gas Lack of skill search and rescue
	Behavioral challenges before the earthquake	Failure to observe safety precautions Placing additional items in the staircase Placing the pot on the edge of the balcony Blockage of the route due to improper parking
	Behavioral challenges during the earthquake	Inappropriate behavior during the earthquake (unthoughfulness, confusion, etc.) Calling emergency services for trivial issues, which delays providing services to people in need.
	Behavioral challenges after the earthquake	Ignoring mild earthquakes and resting in an unsafe place Failure to observe safety precautions in the tent during emergency accommodation Creating traffic on the streets Emotional behavior and unnecessary travel to the affected area
Challenges related to the physical environment	Building (structural factors)	The impossibility of reducing structural vulnerability in rented houses Buildings are not resistant Building non-standard and illegal houses on the outskirts of the city Failure to observe engineering principles in construction
	Furniture and appliances (non-structural factors)	The impossibility of reducing non-structural vulnerability in rented houses Lack of securing home appliances such as shelves, chandeliers, etc. Worn out non-standard home appliances such as oven, stove, water heater, heater, etc. The house is not safe due to improper wiring and plumbing, etc. Improper arrangement of home appliances and furniture Lack of emergency stairs in most buildings
	Emergency equipment	Lack of emergency accommodation supplies such as tents, etc. (Failure to evacuate the house) Lack of access to special equipment for the disabled Lack of essential manual rubble collection equipment Insufficient home equipment and supplies, such as first aid box, etc.
	Physical texture of the neighborhood	Buildings in the neighborhood lack resistance The narrowness of the passages Apartment living and high population density
	Infrastructure	Lack of access to essential services and equipment in some areas Electricity, gas, and telephone were cut off during the earthquake.
	Weather factors	Emergency accommodation is impossible due to cold weather and rainfall. As a result of failing to evacuate the house at the time of the earthquake The possibility of secondary hazards due to the use of cold season heating machines
Challenges related to the social environment	Security	Failure to evacuate home due to insecurity and possibility of robbery from home Failure to observe others' privacy during emergency resettlement Failure to consider a suitable and secure place for emergency evacuation
	Communications	Inadequate communication with relatives, neighbors, etc., due to lifestyle Inadequate communication between family members Social isolation of vulnerable groups (elderly, disabled, etc.)
	Participation	Non-participation of residents for preventive measures and neighborhood preparation The discouragement of the residents due to the non-cooperation of some members of the neighborhood It is less possible to participate in neighborhood events in big cities.
Challenges related to financial factors	Financial resources	High cost of livelihood and lack of funding for preventive measures and preparations High cost of retrofitting buildings The expensive cost of renting safe houses
	Insurance	Lack of importance of building insurance Lack of financial power to pay insurance

### Challenges related to cognitive factors

#### Lack of knowledge

The participants stated that acquiring knowledge can effectively improve households' preparedness behavior and reduce losses and casualties, but for various reasons, most people's awareness is not enough or is incorrect. For example, A 34-year-old bachelor's degree woman said:

I *am an employee, and when I come home in the evening, I am busy with housework. I have to take care of my husband and children. I don't have time to participate in Red Crescent classes “(P13)*.

#### False beliefs

Participants stated that some people have false beliefs, which lead them to not take preventive and risk reduction measures and feel weak against hazards. A 45-year-old bachelor's degree man who had experienced the Bam earthquake said:

“*Some people think that an earthquake is God's punishment and we can't do anything. Were the children killed in the Bam earthquake because of God's anger? What did they do? The children were innocent. You have to inform the people”* (p5).

#### Self-efficacy

Some participants mentioned that they thought they could not take preventive measures and prepare and behave efficiently when an earthquake occurs. A 52-year-old master's degree woman said:

“*I get a lot of stress during an earthquake. I'm not sure if one day there will be a strong earthquake in Kerman, I will be able to keep my calm” (P4)*.

Some people stated that they do not have the necessary skills to prevent secondary hazards and reduce harm.

A 43-year-old diploma woman said:

*I haven't been trained because I don't think I can help the wounded. If I see someone injured, I feel bad. Someone who chooses to do this must be very brave. I really don't have any skills” (P25)*.

#### Negative outcome expectations

The participants commented that they thought preparing for earthquakes and doing drills at home might harm vulnerable groups' physical and psychological health.

A 37-year-old woman who had a seminary education said:

“*I have three small children. They may not understand why I am preparing a rescue kit, and it scares them because they think something is going to happen” (P1)*.

A 34-year-old master's degree man said:

“*My wife is pregnant; I know she can't move fast. If we try to drill at home, she may fall and get more injured, or she may get stressed, and her blood pressure will rise. We prefer not to drill while my wife is in this condition ”(P15)*.

### Challenges related to behavioral factors

#### Lack of skills

Some participants mentioned that after the devastating Bam and Zarand earthquakes, they had to search for and save their loved ones themselves in the early hours before the rescuers were present. They mentioned that they had no training and did not know how to save an injured person. A 50-year-old bachelor's degree man said:

“*In the Bam earthquake, the whole city was completely destroyed, and there was not enough relief force. We had to pull the families out from under the rubble. People did not know how to save a wounded person. Later, I heard that many of the wounded were paralyzed because we did not know what to do. Everyone, in my opinion, should learn these skills before the earthquake”(p11)*.

#### Behavioral challenges before the earthquake

Participants said that some people's inappropriate behavior causes others to get hurt during an earthquake. A 43-year-old master's degree woman said:

“*When you look at our building, you see that they put flowerpots and other things on the balcony without any protection, so if there is an earthquake, they will fall on people's heads. I wish someone would warn them that this is dangerous” (p33)*.

#### Behavioral challenges during the earthquake

Excessive emotional or unthoughtful behavior is one of the challenges during earthquakes that participants mentioned. A 35-year-old master's degree man said:

“*Many people get fearful and confused. For example, my sister is terrified of earthquakes and does things that are not appropriate at all. When she is so afraid, her children get afraid as well ”(P16)*.

Another challenge that participants said was that some people crowd medical centers for minor injuries that do not require hospital visits.

A 41-year-old bachelor's degree woman who worked in a hospital said:

“*During an earthquake, even with minor injuries that do not require immediate treatment, people call the emergency medical center or go to hospitals, which may cause acute cases to receive delayed medical services” (p43)*.

#### Behavioral challenges after the earthquake

Participants commented that many secondary hazards and challenges after an earthquake can be prevented by preventing emotional and hasty behaviors and following safety tips. A 39-year-old master's degree man said:

“*After the earthquake, people come to the street by car, and there is a lot of traffic in the city. I think they should be taught in advance not to have these behaviors ”(P18)*.

A 48-year-old master's degree woman who had experienced the destructive Bam earthquake said

*After the destruction of Bam city in 2003, many people from far and near cities entered Bam city. Iranians are very altruistic and like to help victims in this situation, but they don't know that this behavior will cause congestion in the city and cause more problems (p14)*.

### Challenges related to physical environment

#### Building (structural factors)

Participants stated that unsafe and non-resistant buildings are among the most challenging.

A 50-year-old diploma man said:

“*In my father's village (*Dahuiyeh*), although the houses were newly built, after the earthquake, 90% of the houses were destroyed, and a large number of people in the village were killed. Because the houses were not built according to engineering principles and standards” (p20)*.

#### Equipment (non-structural factors)

Participants commented that equipment inside buildings can result in death or injury if it falls, breaks, or blocks exit routes.

A 44-year-old physician woman said:

*As a tenant, I can't do many things, I can't even hammer a nail into the wall. The owner of the house may not agree that I fix the shelf so that it doesn't fall down during an earthquake* (P2).

A 43-year-old diploma woman said:

“*Today's houses are very small; we don't have enough space for furniture. We have to put cupboards and shelves in the hall and put many things inside. If an earthquake happens, even if the house is not damaged, these utensils will cause problems for us” (P25)*.

#### Emergency equipment

As some participants commented, appropriate equipment was not available to rescue people. A 50-year-old diploma man, who was severely injured in the Zarand earthquake, said:

“*I was under the rubble for several hours. I could not move. People did not have shovels and picks, and they could not save me. If they had pulled me out from under the rubble earlier. God helped me to survive, but it took me some time to recover” (P20)*.

The participants who experienced the Bam earthquake also stated that one of the main problems in the early hours after the earthquake was the lack of manual debris removal equipment. They believed this equipment should be prepared in advance.

#### The physical texture of the neighborhood

Participants commented that the considerable distance between the houses and the evacuation place, the lack of strength of the buildings in the neighborhood, the density of the buildings, and the insufficient width of the routes could cause many casualties and injuries during an earthquake.

A 48-year-old master's degree woman said:

“*Take a look at our neighborhood and you will notice that the houses are old and dilapidated. The alleys are narrow. If a strong earthquake happens, the houses will crash into each other and the alleys will be closed. My friend said that when I went to Bam after the earthquake, I could not find my father's house because the houses were crashing on each other. Several thousand people died in Bam because they could not save people quickly” (P8)*.

#### Infrastructure

Participants commented that poor infrastructure, lack of access to facilities in some areas, and disruption of communication systems during an earthquake are critical challenges.

A 46-year-old bachelor's degree woman said:

“*When there is an earthquake here, sometimes the phones are cut off. My daughter, who is studying at a university in another city, gets very worried about us if she can't talk to me. The authorities should solve the telecommunications problems. If there is an earthquake, the phones will be cut off, and this causes people to worry because they cannot call [their family members] ”(P37)*.

#### Weather factors

Many participants said that cold weather and rain are significant barriers to an emergency evacuation.

A 41-year-old bachelor's degree woman said:

“*I remember in 2018 or 2019, there were many earthquakes in Kerman, and the weather was cold. Where should we go if we wanted to stay out of our buildings? Has the government considered a place where people could take refuge during an earthquake? We cannot sleep in the park or on the street in winter. Our children get sick ”(P31)*.

### Challenges related to the social environment

#### Security

Participants mentioned that one of the most critical issues during an earthquake is people's security.

A 51-year-old bachelor's degree man said:

“*During an earthquake, people are afraid of their houses being robbed. That is why they prefer to stay in their dilapidated houses and do not leave the houses to go to a secure place” (P3)*.

A 60-year-old primary education woman said:

“*My husband is dead and I don't have a son. If there is an earthquake and we want to leave our house, I can't go and sleep in a park with two daughters. I don't dare. We may be persecuted” (P47)*.

#### Communications

Participants mentioned that more communication between family members, relatives, and neighbors can increase mental preparedness, and knowledge sharing. Insufficient social communication was mentioned by several participants.

A 52-year-old master's degree woman said:

“*People are very busy. They have less time to visit each other. I don't see my relatives often because they live in another city. Also, since I am an employee, I rarely see my neighbors. I think that if we have a lot of communication with each other, we will be aware of each other's problems and we can help each other to solve them. We can also learn a lot from each other about earthquake prevention and preparedness” (P4)*.

#### Participation

The participants mentioned the insufficient collaboration of the neighborhood residents in the fields of preventive measures, reduction of damage, and improvement of preparedness as one of the main challenges for earthquake preparedness.

A 50-year-old bachelor's degree man said:

“*We have been living in this neighborhood for a year. We don't know our neighbors, and everyone is busy with their lives. I remember when there was an earthquake in the area around Zarand. We were living there. During the earthquake, the wall of one of the neighbors got many cracks. We talked to the neighbors and repaired the wall. Because it may fall on the street and harm the people passing by” (P29)*.

### Challenges related to financial factors

#### Financial problems

One of the barriers most participants mentioned was financial difficulties. A 51-year-old diploma man said:

“*Now, the cost of living in Iran is very high. Our income is also very low. We can't even buy a smartphone for our children so that they can study during this Corona situation. I think we should let's spend our money on more important things, not on an earthquake that might not happen at all*” (P23).

A 32-year-old diploma man said

“*My house is made of clay and mud. I know how dangerous it is to live in this house. But I have no choice, I have to live in this house*. *Because building a durable house requires a lot of money, which I don't have” (p24)*.

#### Insurance

One of the barriers most participants mentioned was insurance problems.

A 40-year-old bachelor's degree man said:

“*Because people do not have enough income, they do not pay much attention to insuring their houses, and if an earthquake happens, in addition to putting their lives in danger, they also lose a lot financially” (P42)*.

## Discussion

This research was conducted to identify the challenges and barriers to households' preparedness against earthquakes from the viewpoint of the residents of Kerman. The analysis of the participant's viewpoints showed many challenges and barriers to preventive measures and the preparation of households against earthquakes. Based on socio-cognitive theory, challenges and barriers were categorized into cognitive, behavioral, and environmental (physical, social, and financial) factors.

### Challenges related to cognitive factors

For various reasons, most participants believed that most households lack sufficient knowledge regarding earthquake preparedness measures. In Najafi et al.'s study (2018), which interviewed 132 heads of households living in Tehran, lack of knowledge and insufficient time were identified as the most important barriers to earthquake preparedness (18). A review study showed that Iranians with a higher knowledge level had a better performance in preparing for an earthquake (23). In Appleby et al.'s study in Romania and Malta (2021), most participants were unaware of disaster preparedness guidelines, and those with more knowledge reported greater preparedness (24). According to Yu et al.'s study in China (2020), people with a higher knowledge level had more disaster preparedness behavior (25). Considering that lack of awareness is one of the most important reasons for inadequate preparation for earthquakes, various educational programs should be designed and implemented considering the audience's conditions to improve households' preparedness behavior.

Some of the participants said that people around them believe that adverse events such as earthquakes are controlled by external factors and that humans have no authority over or ability to overcome them. Some participants did not agree with this. These beliefs are considered barriers to health-related behaviors. In a study by Askarizadeh et al. in Tehran, decision-making about risk reduction behaviors decreased with increasing sources of external control (26). According to a study by Chen et al. in China (2019), households with less determinism were more likely to adopt emergency preparedness behaviors (27). In Armas et al.'s study in Romania (2018), people with a belief in an external locus of control had high stress and more worry about hazards, and felt less prepared (28). According to a study conducted in Saudi Arabia (2016), contrary to expectations, households with a higher level of attributing earthquakes to supernatural factors were more prepared for earthquakes. The authors stated that in Muslim societies, based on hadiths and the Qur'an, people may believe that they are obligated to protect their lives from dangers and that any disaster they face results from their behavior (29). Perhaps due to the unpredictability of an earthquake's exact time and place, people may think they have less control over earthquakes and show less preparedness.

One of the challenges raised by the participants was a lack of belief in their abilities for preventive measures and preparation. According to Bandura, self-efficacy is the most important predictor of behavior change. If people believe they cannot perform a behavior, they are not motivated to act or resist challenges and obstacles (17). Studies have shown that the higher a person's self-efficacy is, the more intention there is for preventive measures and preparedness against disasters. In a review conducted by Ranjbar et al., Iranians who had more self-efficacy reported more earthquake preparedness behaviors (23). In Ning et al.'s study in China (2021), people with higher self-efficacy had more emergency preparedness behavior (8). In a study by Greer et al. in the United States (2020), it has been shown that the higher the self-efficacy of individuals, the higher their intention to prepare (30). People are likely to engage in preventive and disaster preparedness behaviors if they are confident in their ability to perform actions. As a result, attempting to improve the skills of households in various fields may lead to a sense of self-efficacy and increased efforts for preventive measures and preparation.

Some of the participants in this study stated that sometimes taking preparedness measures at home may negatively affect their family members, especially vulnerable groups. Therefore, they prefer not to prepare their family members for an earthquake to avoid physical and mental damage. According to the social-cognitive theory, if people believe that their actions to promote health will have more negative consequences than positive ones, they will stop that behavior (17). In Najafi et al.'s study, some of the interviewees stated that the main disadvantage of preparing for an earthquake is creating anxiety in family members (18). In studies conducted by Kelly and Ronan in Australia and New Zealand (31), Azim and Islam in Saudi Arabia (29), and McIvor et al. in New Zealand (32), negative outcome expectations were a negative predictor of disaster preparedness. Therefore, interventions focusing on informing people about the importance of preparedness and its benefits for vulnerable groups and their families should be prioritized.

### Challenges related to behavioral factors

In this research, the participants believed that the lack of necessary skills makes households unable to take the necessary and appropriate measures to save their lives or others in the event of an earthquake. In the study of Ning et al. in China, people who had the skill to do drills and did them regularly were more prepared (8). One important stage of preparation is acquiring the necessary skills, such as first aid, search, rescue, firefighting, drills, etc. Therefore, to improve households' preparedness level, it is necessary to focus on increasing their skills. And in every household, at least one person should learn the mentioned skills.

The interviewees believed that the behavior of most people before, during, and after the earthquake was inappropriate. They were concerned that their emotional behavior during an earthquake would cause more harm to themselves and others. And they stressed that people's behavior should be corrected with proper training before the earthquake.

The participants stated that one of the people's behaviors during an earthquake is to go to the earthquake-affected areas, which can cause many problems, including more damage to the injured, disruption of aid delivery, etc. According to the participants, the presence of volunteers that do not have the necessary skills is one of the most critical challenges during an earthquake. In the Sharifi et al. study, many volunteers entered the area spontaneously after the Kermanshah earthquake. Some volunteers had not even received specialized training in rescuing and transporting the injured, which increases the possibility of injuries in the rescue process (20). Volunteers can be an excellent opportunity to overcome problems in many situations, but volunteers can also create challenges and problems if they are not properly managed and organized.

### Challenges related to physical environment

One of the crucial challenges raised by most of the participants was the building of unsafe and non-resistant buildings. One of the most important basic measures to reduce the vulnerability and preparedness of households against earthquakes, especially in areas with high seismic risk, is the construction of standard and resistant houses. The results of various studies show that residential buildings in Iranian cities are dilapidated. For example, the results of Rezaei and Nouri's study (2017) showed that households in Kerman city are vulnerable to earthquakes in terms of building strength (33). In the study by Armas et al. in Romania (2018), 82% of residents believed that an earthquake was likely; however, only 3% believed their building would not suffer significant damage following a major earthquake (28).

Based on the interviews, some participants believed that other preparation measures are useless when the building is not resistant. In the study of Tekeli-Yeşil et al. in Turkey, households that were sure of the resistance of their building were more prepared, and their information was more about earthquake preparedness measures (19).

Considering that the most essential measure to reduce the vulnerability of households, especially in areas with high seismic risk, is the construction of standard and resistant houses, special attention should be paid to this issue (34). For example, construction should be monitored, and non-standard and illegal houses should be prevented from being built in the suburbs. Also, households with dilapidated houses should be encouraged and supported for renovation or reconstruction.

One of the barriers mentioned by the households was non-structural factors. Although building features are the most critical factor in earthquake mortality, even in the safest structures, if the furniture, equipment, and appliances inside the building are not safe, death and injury can occur. Some participants stated they faced barriers, including living in rental housing, to non-structural vulnerability reduction measures. Other studies showed that homeowners were more prepared for disasters (9, 16, 35). This is probably because homeowners have greater freedom of action to reduce structural and non-structural vulnerability or enjoy a higher socio-economic level. Since most non-structural vulnerability reduction measures, such as securing, immobilizing, moving, proper arrangement of furniture, etc. can be improved with minimal cost and facilities, this challenge can be overcome by designing educational programs for households.

The lack of manual and basic debris removal equipment was a significant problem raised by the Bam and Zarand earthquake survivors. Most participants thought that emergency equipment only included the first aid box. Only those who had previously experienced a devastating earthquake considered manuals and tools necessary for removing debris, in addition to other equipment. Therefore, drawing on the experiences of those who experienced destructive earthquakes can be very helpful.

Some of the participants believed that the narrowness of the roads in the city center, where most of the buildings are not resistant to earthquakes, is one of the critical challenges that must be solved before an earthquake occurs. In areas where the houses are dilapidated and the roads are narrow, in the event of an earthquake, rescue vehicles can't travel, and rescue forces can only provide services with a delay, which will increase casualties and serious complications among the injured. Studies have shown that cities in most regions of Iran are highly vulnerable to earthquakes. For example, in Mesri Alamdari et al.'s (36) study, a significant percentage of Varzeqan city was vulnerable to earthquakes. High residential and population density, low-quality buildings, and a lack of urban open spaces have caused this region to have a severe vulnerability to earthquakes (36). In the study of Salehipour Milani et al. (37), most Razan neighborhoods were close to 55% in the high and very high-risk range. The weak structure of the buildings, the age of the buildings, the narrowness of the roads, and especially the population density, were important factors affecting the city's vulnerability (37). Knowing weak and sensitive areas to earthquakes is the first step in reducing vulnerability to earthquakes and optimizing urban spaces. Therefore, it is necessary to identify vulnerable areas in every city and take urgent action to remove obstacles and problems.

### Challenges related to the social environment

The participants believed that one of the most critical issues during an earthquake is providing security by the police and security forces. They believe that if people do not feel secure, they will not evacuate their homes during an earthquake warning. The evacuation order may cause chaos in the city, and there is a possibility of robbery if the house is evacuated. Also, vulnerable groups, including women and young girls, do not have the possibility of staying outside the house due to the possibility of causing harassment. In a study by Newnham et al. in Hong Kong, many participants mentioned fear of home burglary and lack of a suitable place to stay as factors preventing evacuation during a warning (10). Therefore, people's security should be ensured in all areas so that they can collaborate in evacuating their homes after the warning.

One of the challenges expressed by the interviewees about the preparation of households before the earthquake was insufficient interactions between family members, relatives, and community members. They believe that communication between people has decreased compared to the past for various reasons. They believed that the more people communicated with one another, the better they could support one another in various fields and help one another overcome obstacles. According to the study by Kim and Zakur, people who had higher levels of social support and more connections with society and organizations were more prepared for emergency situations related to disasters (38). A study conducted by Yong and Lemyre on Canadian households showed that receiving social support plays a role in disaster preparedness behavior (39). According to Kanakis and McShane's study conducted in a rural community in Australia that was prone to floods and storms, the higher the perceived social connections, the higher the disaster preparedness behavior of households (40). Social connections can contribute to knowledge sharing, financial support, emotional support, etc. When people are in contact with each other and support each other, they give a faster and more appropriate response to an emergency and recover and rehabilitate faster after disasters.

The non-participation of residents in preventive measures and preparations against earthquakes in the neighborhood was one of the other challenges. In the study of Hadafi et al., in Iran (2021), the participation rate of citizens was at a relatively favorable level, and most of the citizens had a lot of participation in voluntary activities to prepare against the risks related to the neighborhood (41). In the study by Becker et al. based on the interviews conducted with households living in New Zealand, the more the households participated in social groups, the more preventive measures and earthquake preparedness they took (42). People who participated in social activities had more intentions to prepare for earthquakes, according to Zaremohzzaieh et al. in Malaysia (2021) (43) and Adhikari et al. in Nepal (2018) (44). According to Kelly and Ronan's study in Australia and New Zealand (2018), people who participated in discussions about current events and participated in social events were more prepared for earthquakes (31). Social connections can contribute to knowledge sharing, financial support, emotional support, etc. When people are in contact with each other and support each other, they give faster and more appropriate responses to emergencies and recover and rehabilitate faster after disasters.

### Challenges related to financial factors

Almost all participants mentioned financial issues as an essential barrier to preparing households against earthquakes. Buying land in safe areas and building a durable and standard house requires a lot of financial resources that most people may not be able to provide. Some of the participants stated that they could not renovate their homes or buy emergency equipment and supplies due to insufficient income and the increase in other living expenses. They preferred to spend their meager income on daily necessities.

Studies have shown that people with high income levels are more prepared for disasters (10, 31). In the study of Rezaei and Nouri, households with a higher socio-economic level had more knowledge, attitudes, and preparedness against earthquakes (33). In Newnham's study in Hong Kong, in people with higher monthly income, self-efficacy was higher and evacuation barriers were lower when hazards occurred (10). Therefore, the authorities' efforts to reduce society's financial vulnerability need to increase their preparedness against disasters.

## Limitations

The interviews were conducted during the COVID-19 pandemic. Some households did not want to participate in the interviews due to the possibility of disease transmission. Some women and most men were working, so the interviews were conducted in the afternoons or on holidays when they were at home.

## Conclusion

Participants in this study expressed several cognitive, behavioral, social, physical, and financial challenges. Many barriers and challenges can be solved by developing and implementing educational programs. Many of these challenges, however, cannot be overcome by households without the assistance of the government and officials. By removing the challenges and barriers, adopting preventive measures, and improving the level of preparedness, it is possible to decrease the casualties after earthquakes.

## Data availability statement

The original contributions presented in the study are included in the article/supplementary material, further inquiries can be directed to the corresponding author.

## Ethics statement

The Ethics Committee of Kerman University of Medical Sciences approved this study. A Qualitative design was employed in 2021. The code of Pajouhan is 400000068 and the ethic approval code is IR.KMU.REC.1400.719. All methods were performed in accordance with the relevant guidelines and regulations; this article does not contain any studies with animals performed by any of the authors. Informed consent was obtained from all individual participants included in the study written informed consent was obtained from individual participants. Confidentiality and anonymity of the participants were ensured by coding of the questioners. Study participants were informed clearly about their freedom to opt out of the study at any point of time without justifying for doing so.

## Author contributions

The study's concept and design were created by ER. The survey was performed by NK. Data analysis and manuscript writing were handled by HF and MN-M. NK oversaw the research and provided critical feedback on the manuscript. The final manuscript was read and reviewed by all authors.

## Conflict of interest

The authors declare that the research was conducted in the absence of any commercial or financial relationships that could be construed as a potential conflict of interest.

## Publisher's note

All claims expressed in this article are solely those of the authors and do not necessarily represent those of their affiliated organizations, or those of the publisher, the editors and the reviewers. Any product that may be evaluated in this article, or claim that may be made by its manufacturer, is not guaranteed or endorsed by the publisher.
